# The risk of developing more potent fentanyl analogs: a mini review

**DOI:** 10.3389/fphar.2025.1723733

**Published:** 2025-11-27

**Authors:** Benjamín Bustamante-Elgueta, Maikol D. Andrade, Cristóbal A. Quintul, Daniel A. Medina, Javier Campanini-Salinas

**Affiliations:** Escuela de Química y Farmacia, Facultad de Ciencias, Universidad San Sebastián, Puerto Montt, Chile

**Keywords:** fentanyl, carfentanil, analogues fentanyl, opioids, new synthetic opioids

## Abstract

The global opioid crisis has been accelerated by fentanyl and its analogues, compounds optimized for potency but burdened by vanishingly narrow safety margins. This mini-review integrates chemical, pharmacological, toxicological, and regulatory evidence to interrogate the “more-potent-is-better” paradigm. We synthesize *in vivo* data across representative analogues, highlighting those compounds that are much more potent than fentanyl and the risks of their use. Moreover, several analogues exhibit markedly low protection indices, indicating that doses producing analgesia lie perilously close to those causing hypoventilation. Reversing the effects of overdose remains pharmacologically feasible, although *in vitro* evidence suggests that antagonists such as naloxone may require higher or repeated doses to counteract ultra-potent fentanyl analogs. Forensic and public-health signals, rapid marketplace turnover, metabolic complexity, polysubstance exposure, and episodic mass poisonings, underscore the risks of continuing to chase potency. We also map the regulatory gap at the health–security nexus and flag dual-use concerns, including AI-enabled design of ultra-potent scaffolds with poor therapeutic windows. We argue for a strategic pivot: prioritize intrinsic safety over potency by targeting wider therapeutic windows, mechanism-level dissociation of analgesia from respiratory depression, standardized antagonist requirements, and class-aware scheduling that preserves legitimate research. Redirecting discovery toward safety-first opioids is both scientifically tractable and ethically imperative.

## Introduction

The emergence of synthetic opioids has redefined the landscape of pain medicine and, concomitantly, has catalyzed one of the most pressing public health crises of our time, the opioid epidemic (Patocka et al., 2024; Skolnick, 2018). Fentanyl and its derivatives, originally designed for therapeutic purposes, have become a determining factor in overdose mortality among young adults in the United States (Leen and Juurlink, 2019). In 2023, there were more than 80,000 opioid-related deaths in the United States; a large majority involved fentanyl or synthetic opioids that are closely related (Obeng et al., 2025).

Fentanyl, a synthetic μ-opioid receptor (MOR) agonist, is notable for its rapid onset of action and a potency approximately 100 times greater than morphine in preclinical antinociception assays (Varshneya et al., 2023). These properties, combined with the simplicity and low cost of its synthesis (which has given rise to over 1,400 derivatives), have favored its rapid global spread (Patocka et al., 2024; Misailidi et al., 2018). Nevertheless, it is this very potency that presents a paradox: while in the clinical setting it enables the management of intractable pain, in the context of illicit abuse, it becomes a lethal factor (Vasudevan et al., 2020). The crisis is further complicated by the constant appearance of new synthetic opioids, many of which are chemically modified fentanyl analogues that may fall outside existing controlled-substance lists in some jurisdictions (Edinoff et al., 2023) and, at times, possess potencies greater than the original compound (Wilde et al., 2019).

The pursuit of more potent opioids for pain management and anesthesia (Obeng et al., 2025; Vardanyan and Hruby, 2014) has led to the development of substances such as carfentanil, which is of the order of 10,000 times more potent than morphine in preclinical assays (Mather, 1983). Although these extreme potencies might seem advantageous from a pure pharmacological perspective, toxicological and epidemiological evidence reveals a dark side; analogues that exhibit an extremely narrow therapeutic margin, causing the therapeutic dose to approach the lethal dose dangerously closely (Higashikawa and Suzuki, 2008). Despite their advantages in analgesia and anesthesia, the clinical use of fentanyl carries significant risks, such as respiratory depression and high interindividual variability, which complicates the establishment of safe therapeutic concentrations and makes dosing challenging (Mather, 1983).

The primary cause of death in opioid overdoses is respiratory depression, a direct side effect of MOR activation (Shafi et al.,2022). Fentanyl analogues activate MOR signaling pathways in brainstem regions involved in respiratory control, including the medulla oblongata and the pons, leading to a decreased sensitivity of respiratory centers to hypercapnia and hypoxemia (Vu et al., 2024). The high lipophilicity and slow dissociation of these drugs from the MOR (Walker et al., 2025) could be factors that contribute to the difficulty in reversing overdoses with naloxone, the opioid antagonist of choice, often requiring higher and repeated doses (Shafi et al., 2022). In this scenario, factors such as the easy portability of these drugs, their high potencies (microgram level), their rapid penetration into the central nervous system, and the existence of legal or emerging precursors/analogues increase the plausibility of use that is detrimental to public health (Misailidi et al., 2018).

Beyond the healthcare sphere, the ease of synthesis and extreme potency of these compounds generate serious international security concerns. Fentanyl derivatives have been identified as potential chemical weapons, especially as incapacitating agents, as occurred in the Moscow theater incident, where these compounds were used (Pitschmann and Hon, 2023; Tin et al., 2021; Gummin, 2020).

The available literature focuses mainly on case reports, descriptions of compounds, and isolated alerts, but lacks a cross-sectional analysis that integrates key pharmacological aspects such as pharmacodynamics in MORs, toxicokinetic relevant to reversal strategies, and regulatory and forensic implications. In addition, there is a marked absence of specific regulatory frameworks for new fentanyl derivatives, allowing the synthesis and dissemination of molecules with no therapeutic value and exclusively harmful potential. This lack of scientific and regulatory integration limits the discussion on the development of more potent opioids, which is currently based more on fragmented assumptions than on consolidated evidence.

Although numerous reviews address synthetic opioids, most remain fragmented, examining pharmacological or regulatory aspects in isolation. This work differs by integrating pharmacodynamic potency, toxicological risk, and regulatory gaps to clarify how these factors converge to intensify the current crisis. Our approach adds value by critically addressing the ongoing drive to develop increasingly potent analogues without adequate evaluation of their safety or therapeutic justification.

The data and information used in this article were collected through searches of the PubMed and Google Scholar databases, using keywords such as fentanyl, fentanyl analogues, opioids, and structure-activity fentanyl analogues. Most of the references correspond to articles published from 2015 onwards, in order to include recent and updated evidence. However, some studies prior to 2000 were included, as certain fentanyl derivatives only have clinical research available from that period. Publications in both English and Spanish were also considered.

## Pharmacodynamics and landscape of fentanyl analogues

**FIGURE 1:**
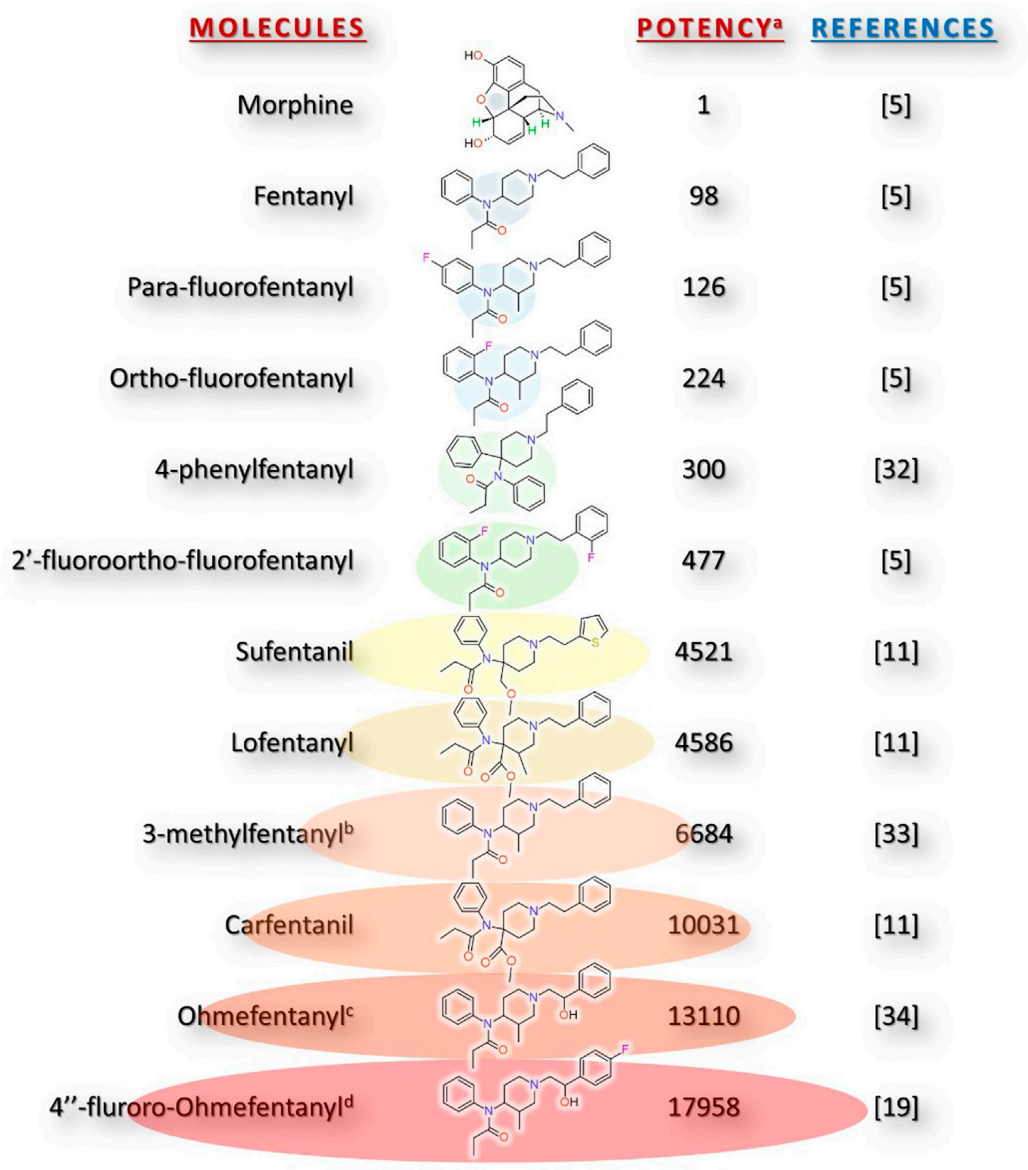
Chemical structures of fentanyl analogues and their relative potency compared to morphine. **(a)** The information regarding relative power presented in this article comes from the values reported in the various studies referenced in this analysis. **(b)** Stereoisomers of (3-S,4-R)-(+)-cis-3-methylfentanyl. **(c)** Stereoisomers of (3R,4S,2′S)-(+)-cis-ohmefentanyl. **(d)** Stereoisomers of (3R,4S,2′S)-(+)-cis-fluoro-ohmefentanyl.

Fentanyl established itself as the prototype of a class of synthetic opioid analgesics, the 4-anilinopiperidines (Mather, 1983). Its primary action is as a selective MOR agonist, with an inhibition constant (Ki) of 0.135 nM, although it also exhibits some affinity for κ-opioid (KOR) and δ-opioid (DOR) receptors, which is much lower compared to MOR (Ki of 174 nM for KOR and 220 nM for DOR). The wide variety of fentanyl analogues has shown potencies even greater than this. Furanyl fentanyl displays a higher affinity for the MOR than fentanyl (Ki of 0.0279 nM), while ortho-fluorofentanyl is 2.67 times more potent in antinociceptive activity (ED50 of 0.03 mg/kg vs. 0.08 mg/kg) (Patocka et al., 2024). The compound 2′-fluoro ortho-fluorofentanyl is notable, being 4 times more potent than fentanyl in antinociception (ED50 of 0.02 mg/kg) (Varshneya et al., 2023). It is interesting to note the stereospecificity of the MOR, with potent derivatives found: 3-methylfentanyl, ohmefentanyl, fluoro-ohmefentanyl, only their specific stereoisomers exhibit high potencies (Figure 1).

The emergence of carfentanil and other ultra-potent fentanyl analogues (Figure 1) (Table 1), such as 4″-fluoro-ohmefentanyl (more potent than carfentanil) (Zou et al., 2003), has been associated with serious health risks, including overdose, respiratory arrest, risk of infectious diseases, severe dependence, and death; with acute toxicities manifesting at microgram levels (Negri et al., 2021). As in the case of Fluorofentanyl, blood concentrations of just nanograms per milliliter to achieve an effect evidence an extremely narrow therapeutic margin (Truver et al., 2022). Furthermore, analysis of the study by Varshneya et al. (2023) indicates that even minimal modifications to the fentanyl scaffold can produce analogues with a markedly increased risk of respiratory depression without a corresponding enhancement of antinociceptive efficacy. This underscores the potential to generate highly toxic fentanyl analogues lacking legitimate therapeutic utility.

**TABLE 1:** Relative potency, ED_50_ of fentanyl analogues (according to the study model and references indicated) and uses (according to references indicated).

Compound name	Potency relative to morphine^a^	ED_50_ (mg/kg)^a^	Study model	Reference Study	Use	Reference Use
Morphine	1	7.8	Warm-water tail-withdrawal tests in rats	Varshneya et al. (2023)	Medical and Drug of abuse	Kiyatkin (2019)
Fentanyl	98	0.08	Warm-water tail-withdrawal tests in rats	Varshneya et al. (2023)	Medical, Drug of abuse and Harm potential	Gerona et al. (2025), ; Weedn et al. (2021), Urbina et al. (2022), Kudzma et al. (1989), Van Bever et al. (1974), Wang et al. (1995), Kiyatkin (2019), Mounteney et al. (2015)
Para-fluorofentanyl	126	0.06	Warm-water tail-withdrawal tests in rats	Varshneya et al. (2023)	Research	Varshneya et al. (2023)
Ortho-fluorofentanyl	224	0.03	Warm-water tail-withdrawal tests in rats	Varshneya et al. (2023)	Research	Varshneya et al. (2023)
4-phenylfentanyl	300	0.011	Hot plate mouse	Kudzma et al. (1989)	Research	Kudzma et al. (1989)
2′-Fluoro ortho-fluorofentanyl	477	0.02	Warm-water tail-withdrawal tests in rats	Varshneya et al. (2023)	Research	Varshneya et al. (2023)
Sufentanil	4,521	0.0007	Hot plate mouse	Mather (1983)	Medical	Cuiabano et al. (2025)
Lofentanyl	4,586	0.0007	Hot plate mouse	Mather (1983)	Previously as a medical and veterinarian	Bilsback et al. (1985), Wootton et al. (1988)
3-methylfentanyl^b^	6,684	0.001	Tail withdrawal test in rats	Van Bever et al. (1974)	Drug of abuse	Hibbs et al. (1991)
Carfentanil	10,031	0.0003	Hot plate mouse	Mather (1983)	Veterinarian and Drug abuse	Vu et al. (2024), Walker et al. (2025), Pitschmann and Hon (2023), Tin et al., 2021; Gummin (2020), Zou et al. (2003), Negri et al. (2021), Truver et al. (2022), Prekupec et al. (2017), Albores-García and Cruz (2023), Zawilska et al. (2021)
Ohmefentanyl^c^	13,110	0.0011	Hot plate mouse	Wang et al. (1995)	Potential drug of abuse	Arillotta et al. (2020)
4″-Fluoro-ohmefentanyl^d^	17,958	0.0008	Hot plate mouse	Zou et al. (2003)	Research	Zou et al. (2003)

^a^ The information regarding relative power and ED, presented in this article comes from the values reported in the various studies referenced in this analysis.

^b^ Stereoisomers of (3-S,4-R)-(+)-cis-3-methylfentanyl.

^c^ Stereoisomers of (3R, 4S, 2′S)-(+)-cis-ohmefentanyl.

^d^ Stereoisomers of (3R, 4S, 2′S)-(+)-cis- fluoro-ohmefentanyl.

The evidence found revealed heterogeneity in the animal model experiments and even in the species used to evaluate the potency of the various analogues (Table 1). To establish a comparison between the most potent fentanyl analogues, only studies in rats and mice were selected.

## Potency versus safety: a clinical dilemma

The most critical adverse effect, and the main cause of overdose death, is respiratory depression (Vasudevan et al., 2020; Shafi et al., 2022), and small structural variations in fentanyl analogs can significantly modify their interaction with the μ-opioid receptor, generating divergent effects between analgesic efficacy and respiratory depression (Varshneya et al., 2023). The safety margin is measured by the protection index (PI), which relates the effective dose causing hypoventilation (ED50) to that producing antinociception. In comparative studies, buprenorphine achieved a PI of 102, morphine of 7.07, and fentanyl of 12, which is higher than that of morphine. However, several analogues exhibit much lower PIs: valerylfentanyl (3.46), 4′-fluorofentanyl (2.74), ortho-fluorofentanyl (2.81), ortho-fluorobutyrylfentanyl (1.64), and para-fluorobutyrylfentanyl (1.73). The 2′-fluoro ortho-fluorofentanyl, despite its high analgesic potency (ED50 of 0.02 mg/kg), presents a PI of 17.6 (Varshneya et al., 2023). These findings highlight the complexity of structure-activity relationships (SAR) and demonstrate that the ability to cause respiratory depression is not directly related to analgesic potency. Furthermore, small changes in the structure of fentanyl have led to the generation of stereoisomers with potencies even greater than carfentanil (Figure 1) (Prekupec et al., 2017).

## Pharmacological challenges in responding to overdoses

The management of new synthetics opioids (NSO) intoxications is complex. Although naloxone effectively antagonizes MOR (Albores-García and Cruz, 2023), preclinical studies suggest that for opioids as potent as fentanyl, higher or repeated doses may be required, in addition to ventilatory support (Walker et al., 2025; Edinoff et al., 2023; Zawilska et al., 2021). Mechanistic studies suggest that there is no true “resistance” to antagonism, but rather that it is dependent on receptor affinity and functional agonist potency (Feas et al., 2024).

In animal models, 0.03 mg/kg of naltrexone failed to completely block the discriminative effects induced by analogues such as 2′-fluoro ortho-fluorofentanyl, ortho-fluorofentanyl, cyclopropyl fentanyl, and 3-furanyl fentanyl (Walker et al., 2025). Accordingly, in theory, naloxone could also require higher or repeated doses to counteract ultra-potent fentanyl analogs, due to the high receptor affinity and prolonged binding of these compounds.

Data from community programs reveal a high presence of fentanyl and analogues in syringes analyzed in Washington D.C. (fentanyl 72.29%, acetylfentanyl 11.44%, para-fluorofentanyl 6.63%, furanylfentanyl 3.61%), with frequent combinations such as fentanyl + acetylfentanyl (12.08%). These findings reinforce the need for harm reduction interventions and strict Schedule I classification to curb the introduction of new fentanyl analogues (Giltner et al., 2022).

## From medicine to the battlefield: opioids as chemical weapons

A historical event of concern about fentanyl analogs is exemplified by the Dubrovka theater crisis in Moscow, Russia, where fentanyl analogs were used dangerously to resolve a hostage situation, causing the death of more than one hundred civilians (Valenzuela-Tapia et al., 2025). The Russian government did not specify the substances used, but a subsequent study showed that carfentanil and remifentanil were detected using liquid chromatography-tandem mass spectrometry (LC/MS/MS) analysis of clothing extracts from two survivors and urine from a third survivor (Leen and Juurlink, 2019; Pitschmann and Hon, 2023; Tin et al., 2021). Both compounds are potent μ-agonists; carfentanil is used for veterinary immobilization at doses on the order of micrograms, and remifentanil has a very rapid onset of action and a short half-life, Additionally, this tragic event demonstrated that these two substances could be aerosolized (Patocka et al., 2024; Misailidi et al., 2018; Tin et al., 2021).

On the epidemiological-forensic level, the extra-hospital circulation of fentanyl analogues has been evidenced by events with attributable lethality. In Alberta (2016), 22 of 343 fentanyl-related overdose deaths involved carfentanil (Leen and Juurlink, 2019); and in Buenos Aires (2022), 20 deaths and 70 hospitalizations were recorded following the consumption of cocaine contaminated with carfentanil, confirming the population risk when these analogues appear outside of controlled clinical contexts (Valenzuela-Tapia et al., 2025). Meanwhile, the study (Gerona et al., 2025) reported the detection of multiple NPS from surveys conducted in traffic accidents on US highways. Of the 1,000 cases analyzed, 290 (29%) were positive for at least one traditional recreational drug (TRD) or an NPS. Among the most frequently identified substances were central nervous system depressants and their co-occurrence with TRDs, with fentanyl being the most detected drug (9 cases), followed to a lesser extent by analogues such as para-fluorofentanyl (4 cases) and acetylfentanyl.

On the operational-strategic level, evidence indicates that the combination of extremely high potency, low mass requirement, and availability of synthesis makes certain fentanyl derivatives attractive to non-state actors for purposes of incapacitation or harm; however, that same potency carries a high probability of lethality and great heterogeneity of effect due to irregular distribution in enclosed spaces and interindividual variability (Valenzuela-Tapia et al., 2025). This reinforces the idea that the combination of the potency of fentanyl and its derivatives poses a potential risk of these agents being used as chemical weapons (Tin et al., 2021).

## Regulation, policy, and new technologies

Currently, no specific category for “non-lethal chemical weapons” exists within the Chemical Weapons Convention (CWC). Furthermore, fentanyl derivatives do not meet the definition of Riot Control Agents (RCAs), as their effects can be lethal at plausible field deployment dosages (Bilsback et al., 1985). This lack of a specific classification highlights a regulatory gap that complicates the control of fentanyl analogues. Experts have consequently advocated for class-wide controls and more robust multilateral frameworks, as measures unilaterally adopted by certain states (China, for instance) have demonstrated limited efficacy in countering rapid, illicit innovation (Weedn et al., 2021). In this context, the class-wide scheduling strategy implemented in the United States since 2018 has shown some success in curbing the emergence of new fentanyl analogues in the market, although it has been questioned for its potential impacts on legitimate research and the debate surrounding its regulatory scope (Weedn et al., 2021). The dual-use nature of synthetic opioids has been underscored by research describing their ambiguous position as both emerging narcotics and potential chemical agents, thereby revealing critical regulatory shortcomings (Valenzuela-Tapia et al., 2025).

To the legal complexity is now added a technological challenge: the use of artificial intelligence in drug design (AI). Although this tool promises to accelerate the discovery of new therapies, it also poses significant risks; with algorithms that can design high-potency compounds but without maintaining an adequate safety profile, increasing the likelihood of generating molecules with narrow therapeutic margins and low toxic doses (Urbina et al., 2022). An added risk comes from the accessibility of these tools, which could facilitate their misuse in the creation of harmful substances.

## Discussion

The analysis of the provided literature reveals the pursuit and development of synthetic opioids of increasing potency, which have significantly amplified the opioid crisis, transforming what is a valuable therapeutic tool into a lethal threat to public health and global security (Obeng et al., 2025; Edinoff et al., 2023). The central tension lies in the fact that the same molecular architecture designed to achieve controlled analgesia in the surgical environment resulted, in compounds like ocfentanil and carfentanil, in substances that outside the clinical realm represent a threat of lethality that is difficult to reverse on a community scale (Misailidi et al., 2018). The existing disproportion between desired potency and intrinsic safety is manifested in the low protection indices (PI) of several fentanyl analogues, which are lower than that of fentanyl (Varshneya et al., 2023).

Although there is no intrinsic resistance to naloxone, *in vitro* assays show that the amount needed to reverse the effects of synthetic opioids varies depending on their potency (Vasudevan et al., 2020), which anticipates a clinical challenge. Although naloxone is confirmed as an effective antagonist even against the most potent compounds, in real-world intoxication scenarios the difficulty persists of administering repeated doses and ensuring timely ventilatory support, especially in contexts of clandestine use or large-scale incidents. This situation highlights the need to move towards integrated, rather than fragmented, strategies that combine harm reduction-oriented monitoring, the establishment of early warning systems in forensic laboratories, and flexible regulatory frameworks that allow a rapid response to the constant emergence of new analogues (Misailidi et al., 2018; Giltner et al., 2022).

However, it is crucial to recognize the limitations of the analyzed studies. Much of the pharmacological and toxicological information on NSOs comes from *in vitro* studies or animal models. The extrapolation of these data to humans is complex and often not direct due to variability between species, strains, and assay methodologies (Prekupec et al., 2017), so the use of the studies mentioned in this review to assess population risk must be done prudently. Furthermore, the lack of clinical studies leaves significant gaps in our understanding of their effects in humans. Evidence in humans comes from forensic case series and narrative reviews, with methodological heterogeneity (matrices, quantification limits, co-substances). Additionally, the scarcity of data on the pharmacokinetics and metabolism of many non-medically used fentanyl analogues also limits a complete understanding of their risk profiles.

To the health, scientific, and ethical problems is added one of a technological nature: AI. Although its advancement promises great benefits, it could also facilitate the design of molecules for dangerous purposes, posing a critical challenge for national and international regulatory frameworks.

Given this landscape, future directions in opioid research must prioritize a fundamental paradigm shift, from an emphasis on potency to intrinsic safety. Concrete approaches are needed that include: Maximizing the therapeutic window, focusing on the identification and development of new compounds with wider PIs, which robustly separate analgesic effects from lethal adverse effects.

- Prioritizing clinical-toxicological studies that quantify naloxone requirements and respiratory depression trajectories specific to each analogue, under standardized designs.
- Developing more effective, faster-acting opioid antagonists with improved pharmacokinetic profiles, capable of overcoming the high potency and persistence of the most dangerous NSOs.
- Conducting more comprehensive *in vivo* SAR studies that systematically evaluate not only potency but also the safety index and adverse effect profiles of new compounds.
- Studying the degree and form of penetration of fentanyl/analogues in at-risk populations.
- Analyzing the risk of new technological tools, such as AI, in the development of this public health crisis.
- Modernize current regulatory frameworks to address the rapid emergence of new synthetic opioids.

The scientific community, together with regulators and public policymakers, has the ethical responsibility to guide opioid research towards a future where the ability to relieve pain is not intrinsically linked to the risk of death.
